# Assessing the Impact of Animal Husbandry and Capture on Anemia among Women and Children in Low- and Middle-Income Countries: A Systematic Review

**DOI:** 10.1093/advances/nmy080

**Published:** 2019-03-11

**Authors:** Nathalie J Lambrecht, Mark L Wilson, Andrew D Jones

**Affiliations:** 1 Department of Nutritional Sciences, University of Michigan, Ann Arbor, MI; 2 Department of Epidemiology, School of Public Health, University of Michigan, Ann Arbor, MI

**Keywords:** livestock ownership, animal husbandry, fish production, anemia, animal source food consumption, infectious disease, zoonotic disease, women, children

## Abstract

Animal husbandry and capture (AHC) may mitigate anemia among women and children by supplying a source of micronutrient-rich animal source foods (ASF), yet may concurrently increase exposure to anemia-inducing pathogens such as *Plasmodium* spp., helminths, and enteropathogens. We conducted a systematic literature review to assess the relation between AHC and anemia among women of reproductive age, school-aged children, and children aged <5 y in low- and middle-income countries (LMICs). We used a 2-stage screening process, in which 1 reviewer searched 4 databases (PubMed, Web of Science, EMBASE, and Global Health) with predetermined search terms for relevant articles. Two reviewers then independently screened studies using a priori exclusion criteria, yielding a total of 23 articles included in the final review. We evaluated evidence from observational studies assessing animal-dependent livelihoods and livestock ownership, and interventions that promoted livestock and fish production. We found little consistency in anemia outcomes across the several AHC exposures and population groups. Poultry production interventions had modest benefits on anemia among women and children, although whether these improvements were a result of increased ASF consumption, or a result of the combined treatment study design could not be determined. Observational studies identified chicken ownership, and no other livestock species, as a risk factor for anemia among young children. However, there was limited evidence to evaluate pathways underlying these associations. Studies tended to rely on self-reported fever and diarrhea to assess illness, and no study directly assessed linkages between AHC, pathogen burden, and anemia. Thus, there is insufficient evidence to conclude whether AHC improves or worsens anemia among women and children in LMICs. Given the current interest in promoting animal production among low-income households, future studies with robust measures of livestock ownership, ASF consumption, pathogen burden, and anemia status are needed to understand the nuances of this complex and potentially contradictory relation.

## Introduction

Animals play an integral role in the livelihoods of people around the world. For the nearly 1 billion rural poor living in Sub-Saharan Africa and South Asia that raise livestock (1) and the approximately 160 million people engaged in fish-related activities (2), animals are a source of income, food, manure, draught power, transportation, financial stability, and social status (3). Animal source foods (ASF) are rich in key micronutrients often missing in staple-based diets, notably iron, zinc, vitamin A, vitamin B12, riboflavin, calcium, and essential fatty acids (1, 3). Inadequacies in these micronutrients contribute to severe short- and long-term health consequences, especially among women and children living in low- and middle-income countries (LMICs) (4). Consumption of ASF is, therefore, a viable strategy for reducing micronutrient deficiencies among women and children. At the same time, animal rearing may have negative repercussions for human health. Raising or consuming animals can expose people to infectious disease pathogens through direct contact, or indirectly via blood-feeding arthropod vectors and fecal contamination of the local environment (5, 6). Such zoonotic infections in humans, which involve microbe transmission cycles among animals and “spillover” to humans, significantly contribute to the human disease burden in LMICs, particularly in resource-poor environments where animals and humans live in close proximity (7).

**FIGURE 1:**
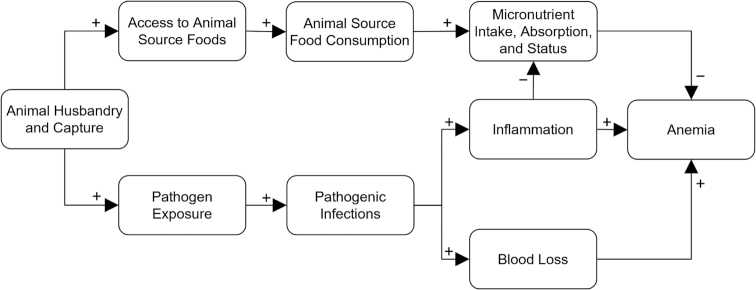
Simplified conceptual framework of the main pathways connecting animal husbandry and capture (AHC) with anemia among women of reproductive age, school-aged children, and children aged <5 y that are assessed in this review. AHC is hypothesized to reduce anemia through animal source food consumption (top pathway) and increase anemia through pathogenic infections (bottom pathway).

This review focuses on anemia, a condition that we hypothesize may be particularly sensitive to both the potential benefits and harms of animal husbandry and capture (AHC), which includes livestock rearing, hunting, and fishing. Anemia affects over 800 million women and young children globally, with the highest prevalences occurring in Sub-Saharan Africa and South-East Asia (8). Children and women are physiologically most vulnerable to developing anemia compared to other populations, and this condition can adversely affect cognitive development, reduce work performance, and increase the risk of mortality among these groups (9). Anemia can result from micronutrient deficiencies, infectious diseases, and genetic disorders (9, 10). Thus, consumption of ASF may mitigate anemia risk by providing a dietary source of heme iron, vitamin A, and vitamin B12 if animals are allocated for consumption directly from own production or via income from AHC (3, 11) (**Figure 1**). On the other hand, engagement in AHC may increase exposure to several anemia-inducing pathogens, including: a) *Plasmodium* species (12) which destroy erythrocytes, suppress erythrocyte production, and induce hepcidin-mediated iron sequestration, manifesting as malaria (13); b) soil-transmitted helminths and *Schistosoma* species (5, 14) which cause blood loss (15, 16); and c) enteropathogenic bacteria (17, 18), hypothesized contributors to inflammatory signals that induce iron sequestration, reduce iron absorption, and reduce erythropoiesis (19) (Figure 1).

To the authors’ knowledge, no previous studies have systematically reviewed the literature on the relation between AHC and anemia among women and children. Previous reviews have examined aspects of this literature, although as part of a broader assessment of the impacts of agricultural programs on diets and nutrition outcomes (20–23). The evidence from these reviews suggests that ASF consumption was often increased among intervention groups that participated in poultry production, dairy development, home gardening, or aquaculture interventions. However, there was insufficient evidence to draw conclusions about the impact of animal production on anemia or other nutritional outcomes because of methodological limitations of studies such as inappropriate control or comparison groups (21, 23), lack of control for confounding bias (21, 23), and insufficient statistical power (22, 23). Yet, understanding the potential for AHC to contribute to anemia (among other health outcomes) is essential, especially given recent efforts to promote small-scale animal husbandry at scale among low-income rural residents (24). New evidence has emerged in recent years regarding the potential for exposure to animals and animal feces to deleteriously affect nutrition and health outcomes, especially among children (25–27). However, the benefits of AHC for diets, livelihoods, and social standing in LMICs may be substantial. A summary of the current state of knowledge on the linkages between AHC and anemia, as well as the complex dietary and infectious disease pathways that underlie these linkages, is needed to inform both a future research agenda and nutrition-sensitive agriculture policies.

Thus, the objective of this systematic review was to assess the relation between AHC and anemia among women of reproductive age, school-aged children, and children aged <5 y in LMICs. Our review builds upon the preceding reviews by including new intervention studies conducted in the last 5 y as well as observational studies not previously reviewed.

## Methods

This systematic review follows the reporting guidelines set forth by the PRISMA (Preferred Reporting Items for Systematic Reviews and Meta-Analyses) statement (28). The review protocol was not registered.

### Definitions

AHC was defined as ownership of animals, exposure to animals, and/or livelihoods related to animal rearing and capture (e.g., pastoralism, transhumance, fishing) for the purpose of producing food, income, farming services (e.g., manure, draught power), serving as savings or insurance, and/or contributing to social status. Animals of interest included domestic terrestrial livestock (e.g., cattle, camels, goats, sheep, pigs, other small ruminants, and poultry/chickens), wild animals, and fish. Insects were not included in this definition. Populations of interest were defined by sex and age ranges: women of reproductive age including adolescent girls and women between the ages of 15 and 49 y and hereafter referred to as “women,” school-aged children including girls and boys between 5 and 14 y, and children <5 y including girls and boys between 0 and 59 mo and hereafter referred to as “young children.” Anemia was defined as low hemoglobin (Hb) concentration, using the cutoffs specified by each study. The WHO recommended cutoffs for anemia are Hb concentration <110 g/L for children aged 6–59 mo, <115 g/L for children 5–11 y, <120 g/L for children 12–14 y, <120 g/L for non-pregnant women, and <110 g/L for pregnant women (29).

### Literature search and study selection

We conducted a systematic review of the peer-reviewed and grey literature from 4 databases (PubMed, Web of Science, EMBASE, and Global Health). One reviewer (NL) searched the databases for relevant articles published through 1 September, 2017. The searches, formatted for each database, included terms encompassing AHC (e.g., livestock, poultry, pastoral, fishing, bushmeat), anemia (e.g., anemia, Hb), and the population of interest (e.g., women, children). The full search strategy used for each database can be found in **Supplemental Table 1**. Although the primary objective was to identify studies examining the relation between AHC and anemia, we also included search terms to identify potential mediating pathways of the relation between AHC and anemia (e.g., animal source food consumption, infectious disease). We also identified studies from previous reviews that assessed the impact of agricultural interventions (including animal husbandry) on nutritional outcomes among women and children (20–23), as well as performed a “forward search” of articles citing these reviews.

Articles retrieved from the searches were screened in a 2-stage process. First, titles and abstracts were screened by 1 reviewer (NL) for potential relevance to the research question. The full texts of potentially relevant articles were then independently assessed for inclusion by 2 reviewers (NL, AJ). Articles agreed upon by both reviewers were included in the review and were eligible for abstraction. Discrepancies between the 2 reviewers were resolved by discussion among all 3 authors.

### Inclusion and exclusion criteria

The full text of each article was screened for study characteristics, study subjects, and exposure and outcome measures. No exclusion criteria were set based on study design, thus both observational and experimental studies were screened for inclusion. Because, to the authors’ knowledge, no previous reviews exist that focus on the effect of AHC on anemia, we did not set any date restrictions. Studies were excluded if the study location was not in a LMIC. Classification of a country's economic status was defined using the World Bank classification system, which categorizes countries by economic status into low-income, lower-middle-income, upper-middle-income, and high-income (30). Studies were excluded if the subject population for which anemia was measured did not include women, school-aged children, or young children as defined above. To be eligible for inclusion, studies had to measure an association between AHC and anemia or Hb. For observational studies to fulfill this criterion, they had to evaluate an effect measure [e.g., odds ratio (OR)] that assessed associations between AHC and anemia or Hb concentration, or a comparison of 1 of these outcomes between populations that differed by AHC livelihoods. For experimental studies, the intervention group(s) must have received an animal-based intervention, and every study had to include an effect measure (e.g., odds ratio, difference in difference) comparing anemia or Hb concentration. We did not exclude intervention studies based on the selection of control group(s), nor did we exclude interventions that combined the animal-based intervention with other agricultural or nutrition interventions (e.g., home gardening, nutrition education, supplementation). Only studies published in English were included in the review.

### Data abstraction and analysis

Data from all studies deemed eligible for inclusion were abstracted into a standardized Excel spreadsheet. The abstracted data included study identifiers, year, country, study design, population characteristics, AHC exposure measure (observational studies) or AHC intervention (experimental studies), intermediate outcome measures from the conceptual framework (i.e., diet, morbidity), and anemia outcome measures. Morbidity was used as an indicator of infectious disease and included any assessment of infection or inflammation, including illness symptoms (e.g., fever, diarrhea), pathogenic infections, and inflammation biomarkers. For the intermediate outcome measures, given our emphasis on AHC as the primary exposure of interest, only studies that provided data for the association of AHC with diet or morbidity, whether or not the association of diet or morbidity with anemia was examined, were included.

We assessed risk of bias within individual studies via an approach modeled after the Grading of Recommendations, Assessment, Development, and Evaluation (GRADE) guidelines (31). GRADE provides both a systematic and transparent methodology for evaluating how well studies in a systematic review support the outcome of interest. Risk of bias assessment evaluates the internal validity of each study to provide evidence for the outcome and does not necessarily reflect the overall quality of the study. For example, in our review, a randomized controlled trial may have been conducted according to the highest standards of rigor yet may be classified as having a “high risk of bias” if the study's main outcome of interest was not anemia. Given that the majority of our studies were observational and quasi-experimental and that the risk of bias criteria developed by GRADE evaluates randomized clinical trials, we modified the GRADE approach using other review papers (22, 32), Strengthening the Reporting of Observational Studies in Epidemiology guidelines (33), the National Institutes of Health quality assessment tool (34), and Cochrane risk of bias criteria (35) to develop our risk of bias categories. Individual studies were evaluated on *1*) counterfactual assessment, *2*) sample size, *3*) anemia outcome assessment, *4*) intermediate outcome assessment, and *5*) confounding bias assessment (**Supplemental Table 2**). **Supplemental Table 3** describes the risk of bias criteria. For each criterion, studies were given a rating of “yes” if they met the criterion, “no” if they did not, or “unclear” if there was not enough information for assessment. An overall assessment of the risk of bias from each study, either low, high, or unclear, was determined based on a weighted judgment of the 5 criteria, with the counterfactual assessment and confounding assessment determined to be the highest priority categories.

Because of the heterogeneity in exposures and reported effect estimates, we did not conduct a meta-analysis to quantitatively assess the evidence from the reviewed articles but instead provide a qualitative narrative of the findings from the included studies. To evaluate the overall body of evidence for our research question, we did not conduct a risk of bias across studies assessment but rather summarized the general direction of anemia outcomes within each AHC exposure from all studies and from studies judged as having “low” or “unclear” risk of bias.

## Results

### Overview of search results and characteristics of included studies

**FIGURE 2:**
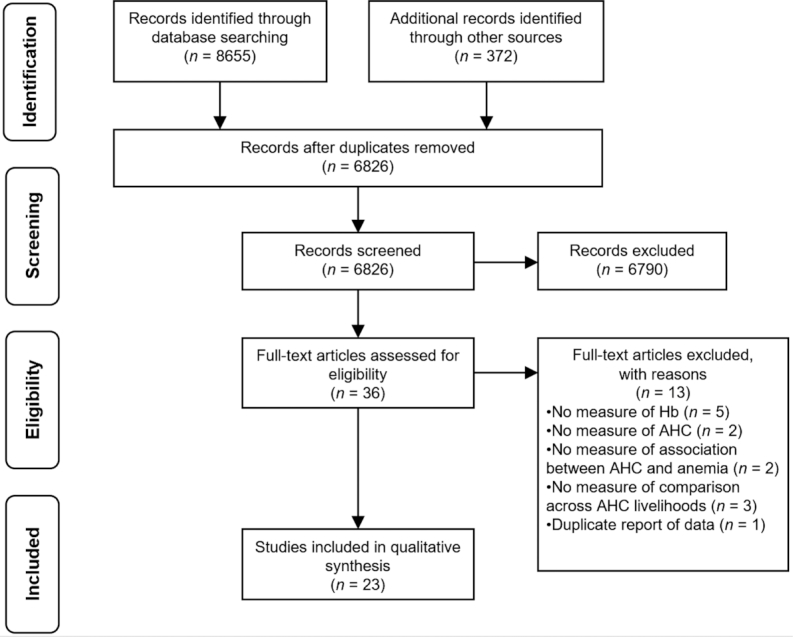
Preferred Reporting Items for Systematic Reviews and Meta-Analyses (PRISMA) flow diagram. AHC, animal husbandry and capture; Hb, hemoglobin.

A total of 6826 unique records were identified through the literature search. Initial screening of titles and abstracts yielded 36 full-text articles. Thirteen of these studies were excluded based on the inclusion and exclusion criteria described above. Thus, a total of 23 studies were included in the final review (36–58) (**Figure 2**).

The included studies were grouped into 3 categories which emerged based on the type of study conducted: *1*) observational studies assessing anemia status between communities or regions with differing AHC-related livelihoods (*n* = 8), *2*) observational studies assessing the association between AHC and anemia (*n* = 6), and *3*) AHC intervention studies (*n* = 9) (**Table 1**). A majority of the studies focused on traditional domestic livestock rearing while 7 included fish, either as fishing or as fish rearing in ponds. Young children and women, but not school-aged children, were the primary populations of interest (over 80% of studies). Most studies (*n* = 16) were cross-sectional in design, and the rest were designed as longitudinal studies (*n* = 4) or randomized controlled trials (*n* = 3). Eleven studies were conducted in the Sub-Saharan Africa region, 11 in Asia, and 1 in Latin America. Almost all the studies examining AHC livelihoods were conducted in Sub-Saharan Africa, whereas all but one of the AHC intervention studies were conducted in Asia. Over half of the studies assessed diet associated with AHC, either as iron intake or ASF consumption, whereas one-third reported on morbidity associated with AHC. The most common measure of morbidity was reported recent illness (e.g., diarrhea, fever).

**TABLE 1:** Summary of studies assessing the association between animal husbandry and capture (AHC) and anemia among women of reproductive age, school-aged children, and children aged <5 y^1^

					Intermediate outcomes		
Author (year, country) (Ref)	Population (sample size^2^)		AHC livelihood, intervention, and/or measure	Anemia outcomes^3^	Diet	Morbidity	Risk of bias^4^
Observational studies assessing anemia status between AHC livelihoods Adongo et al. (2012, Kenya) (36)	Non-pregnant WRA, 15–49 y (*n* = 99/224)	Cross-sectional	Semi-settled vs. settled pastoral communities; TLU	Lower Hb in semi-settled (10.7 g/dL) vs. settled (11.6 g/dL)* Higher IDA among semi-settled (25%) vs. settled (15%) TLU not correlated with nutritional status	Lower iron intake in semi-settled (10 mg) vs. settled (13 mg)***	Higher AI among semi-settled (36%) vs. settled (24%). CRP/AGP NS different	Unclear
Agyepong et al. (1997, Ghana) (37)	Adolescent girls, 10–19 y (*n* = 213/227)	Cross-sectional	Fishing vs. farming community	Higher Hb in fishing (12.5 g/dL) vs. farming (10.8 g/dL)*** Lower anemia among fishing (16%) vs. farming (79%)	N/A	Lower malaria among fishing (29%) vs. farming (47%)**	High
Bechir et al. (2012, Chad) (39)	Pregnant and non-pregnant WRA (*n* = 126/221); Children, 0–59 mo (*n* = 215/398)	Cross-sectional	2 mobile pastoralist groups vs. sedentary community in shared pastoral zone	Higher anemia among pastoralist non-pregnant women (41%, 37%)/children (32%, 28%) vs. sedentary women (26%)/children (23%) (DNS)	N/A	Intestinal parasite infection NS different among pastoral women (86%, 68%) vs. sedentary (74%)/pastoral children (72%, 60%) vs. sedentary (62%) Low malaria among all groups	High
Dalsin (2002, Kazakhstan) (41)	Non-pregnant and non-lactating WRA, 15–49 y (*n* = 239/270)	Cross-sectional	3 livestock-keeping regions; HH production of (cattle, goats, horses, pigs, sheep, poultry); HH engaged in (fishing, hunting)^5^	Higher anemia among southern sheep/goat (46%) and northern cattle/poultry/fishing region (40%) vs. central cattle (29%) region	Central HHs consumed less meat/more dairy Northern HHs consumed more poultry	N/A	High
Jankowska et al. (2012, Mali) (45)	Children, 6–59 mo (*n* = 14,238) grouped into clusters (*n* = 407)	Cross-sectional (secondary data set)	8 livelihood zones (pastoral, rice, plateau, millet, rainfed millet, south crops, remittances, urban)	Livestock livelihoods assoc. with lower anemia (pastoral nomadic***, transhumance and, rice*)	N/A	N/A	Unclear
Keverenge-Ettyang et al. (2006, Kenya) (47)	Pregnant (*n* = 250)/lactating WRA (*n* = 223)	Longitudinal (third trimester pregnancy and 4-mo post-delivery)	Pastoral vs. farming communities	Lower Hb among pastoral (119 g/L) vs. farming (124 g/L) in third trimester* Higher anemia among pastoral vs. farming during pregnancy (42.2% vs. 21.8%**)/lactation (43.4% vs. 27.7%*) Higher ferritin in pastoral vs. farming during pregnancy (25.8 µg/L vs. 24.4 µg/L*)/lactation (18.5 µg/L vs. 16.9 µg/L**)	N/A	N/A	High
Miller (2010, Kenya) (49)	Lactating WRA (*n* = 200)	Cross-sectional	Pastoral vs. 2 mixed pastoral-subsistence agriculture communities; livestock units	Higher Hb among pastoral (13.4 g/dL) vs. mixed pastoral-subsistence (12.7 g/dL) Lower anemia among pastoral (20.6%) vs. mixed (31.8%) Living in pastoral community assoc. with higher Hb** Livestock units NS predictor of Hb among all women	N/A	Lower reported illness (fever, diarrhea, or respiratory infection) in pastoral (25.0%) vs. mixed (48.5%)	Low
Nathan et al. (1996, Kenya) (50)	Children, <6 y (*n* = 104/174)	Cross-sectional (within longitudinal study)	Nomadic pastoral vs. sedentary agro-pastoral vs. sedentary peri-urban	Higher Hb in nomadic (11.03 g/dL) vs. sedentary agro-pastoral (10.83 g/dL) and peri-urban (9.47 g/dL**)	Higher milk/lower meat consumption in nomadic pastoral vs. sedentary communities	Days reported illness (diarrhea, fever, or respiratory infection) NS different between communities	Unclear
Observational studies assessing associations between AHC and anemia Ahenkorah et al. (2016, Ghana) (38)	Pregnant WRA (*n* = 400)	Cross-sectional (200 anemic WRA, 200 non-anemic WRA)	Presence of domestic livestock	Keeping domestic livestock assoc. with higher anemia. (aOR: 2.15, 95% CI: 1.33, 3.68**)	N/A	N/A	Low
Custodio et al. (2008, Equatorial Guinea) (40)	Children, <5 y (*n* = 523/552)	Cross-sectional	Ownership of ≥1 domestic animal (pig, hen, or goat); hunting by a HH member; fishing by a HH member	HH chicken ownership assoc. with lower moderate to severe anemia (DNS) Hunting assoc. with higher moderate to severe anemia** (DNS)	N/A	N/A	High
Flores-Martinez et al. (2016, Afghanistan) (42)	Pregnant and non-pregnant WRA, 15–49 y, (*n* = 9174)	Cross-sectional (secondary data sets)	HH ownership of cattle; horses/donkeys; goats; sheep; chickens	HH sheep ownership assoc. with lower anemia in altitude-adjusted and -unadjusted logistic regression models (aOR: 0.830, 95% CI: 0.73, 0.94**; 0.802, 95% CI: 0.69, 0.93**) HH chicken ownership assoc. with higher altitude-unadjusted anemia (aOR: 1.193, 95% CI: 1.04, 1.36**) HH goat ownership assoc. with lower altitude-adjusted anemia (aOR: 0.839, 95% CI: 0.74, 0.95**) Ownership of any other animal NS assoc. with anemia in either model	Higher mutton consumption in sheep-owning HHs (aOR: 1.27, 95% CI: 1.15, 1.42**) HH sheep ownership increases likelihood of consuming mutton more d/wk** and consuming a greater weight of mutton**	N/A	Low
Iannotti et al. (2015, Haiti) (44)	Children, 3–13 y (*n* = 1047/1167)	Longitudinal, within cluster RCT	Poultry ownership	Poultry ownership assoc. with higher severe anemia in adjusted logistic regression model (aOR: 2.06, 95% CI: 1.02, 4.15*)	N/A	N/A	Unclear
Jones et al. (2018, Ghana) (46)	Non-pregnant WRA, 15–49 y (*n* = 4441); Children, 6–59 mo (*n* = 2735)	Cross-sectional (secondary data sets)	HH livestock ownership; HH ownership of cattle; horses/donkeys/mules; goats; pigs; rabbits; grasscutter; sheep; chickens; other poultry	HH livestock ownership not assoc. with anemia in women (aOR: 1.0, 95% CI: 0.83, 1.2), but assoc. with higher anemia in children (aOR: 1.5, 95% CI: 1.1, 2.0**) HH chicken ownership assoc. with higher anemia among children (aOR: 1.6, 95% CI: 1.2, 2.2**) Ownership of any other animal NS assoc. with anemia in women or children	HH livestock ownership NS assoc. with recent ASF consumption in children HH chicken ownership assoc. with higher HH consumption from own-produced chicken meat*** and eggs*	N/A	Low
Schipani et al. (2002, Thailand) (55)	Children, 1–7 y (*n* = 60)	Longitudinal (matched on fish pond; 3 time points: rainy, cool, hot season)	Mixed-gardening (fishpond, small-animal husbandry, vegetable garden, fruit orchard) vs. nongardening	Hb NS different in mixed-gardening (e.g., rainy: 11.8 g/dL) vs. nongardening children (11.6 g/dL) at all time points Ferritin higher in nongardening vs. mixed-gardening	NS difference in daily iron intake	N/A	Unclear
AHC intervention studies on anemia outcomes Hillenbrand and Waid (2014, Bangladesh) (43)	Children, 6–59 mo (*n* ∼2400); Mothers (*n* ∼2400) Note: Sample size undefined	Repeated cross-sectional with comparison group	Combined poultry rearing, home gardening, and nutrition education; goat asset-transfer to most in-need beneficiaries	Greater decrease in anemia among children from beneficiary (−41.1pp) vs. comparison communities (−4.5 pp) Decrease in anemia among women from beneficiary communities (−28.7 pp) vs. increase in comparison communities (+12.0 pp)	Increased egg production and consumption in children and women^6^	N/A	Unclear
Kumar and Quisumbing (2010, Bangladesh) (48)	Women, >15 y (*n* = 350) Note: *n* = 1237 HHs at endline	Longitudinal with matched comparison group	Improved vegetable production, group-operated polyculture fishponds, privately operated polyculture fishponds	Reduced anemia among women receiving private fishpond (DID: −29.5pp*) and group fishpond (DID: −8.2 pp) vs. comparison group, minimal change in vegetable intervention (+0.2 pp) Higher Hb among women in all groups (private: +0.01 g/dL, group: +0.235 g/dL, vegetable: +0.035 g/dL)	NS change in total iron consumption by women in all groups	N/A	Low
Olney et al. (2009, Cambodia) (51)	Children, <5 y (*n* = 204/500 at baseline, *n* = 210/500 at endline); Mothers (*n* = 186/500, 179/500)	Repeated cross-sectional with comparison group	Combined small animal production, home gardening and nutrition education	Lower Hb at endline vs. baseline among intervention (10.1 g/dL vs. 11.4 g/dL) and control children (9.8 g/dL vs. 11.1 g/dL), and intervention (11.2 g/dL vs. 12.3 g/dL) and control mothers (11.0 g/dL vs. 12.3 g/dL) Higher anemia at endline vs. baseline among children in intervention (50.5% vs. 16.7%) and control groups (55.6% vs. 21.9%)	Increased egg consumption among women and children* in intervention group	Lower fever among children in intervention group (−10.3pp) vs. control group (+6.5 pp) NS difference in diarrhea among groups	Unclear
Olney et al. (2015, Burkina Faso) (52)	Children, 3–12.9 mo at baseline (*n* = 1331/1452)	Cluster-RCT	Combined poultry rearing, home gardening, and nutrition/anemia education (via OWL or HC members)	Higher Hb among children in intervention villages (OWL DID: +0.24 g/dL; HC +0.51 g/dL) vs. controls Among subset of children (3–5.9 mo at baseline), higher Hb and reduced anemia in HC villages (Hb DID: +0.76 g/dL*; anemia DID: −14.6pp*) vs. control	Children who met minimum dietary diversity criteria in past 24 h vs. those who had not more likely to have consumed milk, eggs, and flesh foods	Diarrhea prevalence reduced in both OWL (−9.8pp*) and HC (−15.9pp*) intervention groups vs. control	Unclear
Osei et al. (2015, Nepal) (53)	Children, 6–9 mo at baseline (*n* = 306)	Cluster-randomized controlled substudy	Combined poultry rearing, home gardening and nutrition education +MNP	Higher Hb in both interventions groups vs. control (DID: +3.6 g/L; +MNP: +4.1 g/L) Lower odds of anemia at endline in intervention groups (aOR: 0.52, 95% CI: 0.25, 1.12; +MNP: 0.69, 0.35, 1.36) vs. control	N/A	Lower diarrhea in non-MNP intervention group vs. control** No differences in fever	Unclear
Osei et al. (2017, Nepal) (54)	Children, 12–48 mo at baseline (*n* = 2106 at baseline, *n* = 2614 at follow-up); Mothers (*n* = 2104/2106, *n* = 2614)	Cluster-randomized controlled study (prospective, nonblinded, multistage)	Combined poultry rearing, home gardening, and nutrition education	Higher Hb among children in treatment (114.3 g/L*) vs. control group (110.8 g/L) at follow-up, but lower in both than at baseline (115.3 g/L vs. 113.6 g/L) Children in treatment group less likely to be anemic (aOR: 0.76, 95% CI: 0.59, 0.98) post-intervention Higher Hb among mothers in treatment (126.5 g/L*) vs. control group (121.9 g/L) at follow-up, but lower in both than at baseline (129.3 g/L vs. 129.6 g/L) Mothers in treatment group less likely to be anemic (aOR: 0.62, 95% CI: 0.48, 0.82) than control group post-intervention	N/A	N/A	Unclear
Talukder et al. (2014, Bangladesh, Cambodia, Nepal, Philippines) (57)	Children, 6–59 mo (*n* ∼1000); non-pregnant mothers (*n* ∼1200)	4 repeated cross-sectional studies with comparison groups	Combined small animal husbandry (mainly poultry), home gardening, and nutrition/health education	Decreased anemia among children in intervention groups (Bangladesh: −19pp***; Nepal: −8pp; Cambodia: −8pp; Philippines: −26pp***), but NS different in intervention vs. control groups Decreased anemia among women in intervention groups in Nepal (−15.1pp*) but NS change in Bangladesh (−6.4pp), Cambodia (−1pp) or among any of the control groups (Nepal: +2.9pp, Bangladesh: +0.3pp, Cambodia: −10.7pp)	Higher chicken liver consumption (among HH) and egg consumption (HH: +3 eggs in last week, mothers: +0.5 eggs, children: +1 egg) among combined Bangladesh/Cambodia intervention groups	N/A	High
Smitasiri and Dhanamitta (1999, Thailand) (56)	School-aged girls, 10–13 y (*n* = 164)	Repeated cross-sectional with comparison group	School-based poultry production, fish ponds, vegetable gardens, education, improved lunches, iron supplementation, and community interventions	Increased Hb in intervention group (+0.3 g/dL) vs. control group (−0.2 g/dL) Increased ferritin in both groups, greater change in intervention vs. control^****^	Increased iron intake in intervention group*, NS change in control	N/A	High
Wang et al. (2000, China) (58)	Children, 0–5 y (*n* = 3474 at baseline, *n* = 2744 at endline)	Repeated cross-sectional, no comparison group	Promotion of home gardens (fruits, vegetables, livestock, poultry, aquaculture) plus young child nutrition education	Lower anemia at endline (12.6%) vs. baseline (61.9%)**	ASF contributed to 4.8% of food consumption in 2–5-y-olds at baseline No endline data	N/A	High

1 AGP, α-1-acid glycoprotein; AHC, animal husbandry and capture; AI, anemia of inflammation (defined as ↓Hb, ↑TfR, ↑CRP/AGP); aOR, adjusted odds ratio; ASF, animal source foods; assoc., associated; CRP, C-reactive protein; DID, difference in difference; DNS, data not shown; Hb, hemoglobin; HC, health committee; HH, household; IDA, iron-deficiency anemia (defined as ↓Hb, ↑TfR); MNP, micronutrient powder; N/A, not available; OWL, older women leaders; pp, percentage points; RCT, randomized controlled trial; Ref, reference; TfR, transferrin receptor; TLU, tropical livestock units; WRA, women of reproductive age.

2 If the number of individuals for which Hb was measured is less than the total sample of individuals, sample size is written as the number of individuals for which Hb was measured/total sample size.

3 Effect estimates, when available, are presented in parentheses with associated significance value (NS *P* > 0.05, *P < 0.05, **P < 0.01, ***P < 0.001, ^****^P < 0.0001).

4 Risk of bias may be judged as low risk, high risk, or unclear risk. Bias assessments reflect author judgments of an article based on the objective of the review and do not reflect the quality of the study, which may have been conducted to investigate a different objective and outcome. Risk of bias assessment is available in Supplemental Table 2.

5 Data obtained from Part 1 of a 2-part series (85).

6 Data obtained from separate report (86).

### Observational studies of AHC livelihoods

There was no consistent relation between community- or regional-level dependence on AHC as a livelihood and individual-level anemia status. Three of 5 studies reported higher anemia among women from AHC-dependent communities, 1 of 3 studies reported higher anemia among young children from AHC-dependent communities, and 1 study reported lower anemia among school-aged children from AHC-dependent communities.

Studies within this AHC livelihood category were of 2 main types. The first type included studies that compared anemia between nomadic and non-nomadic pastoralists residing in the same geographic region. We interpreted more mobile pastoralists as the group more dependent on and “exposed” to AHC. Adongo et al. (36) found that semi-settled (nomadic) Gabra pastoral women in Kenya had almost double the prevalence of anemia compared with their settled (non-nomadic) counterparts. The average number of livestock in semi-settled households was approximately twice that in settled households but was not correlated with nutritional status. Adongo et al. (36) was the only study in this review that assessed the inflammatory biomarkers C-reactive protein and α-1-acid glycoprotein. In combination with measures of iron status (i.e., transferrin receptor and ferritin), they found that semi-settled women had both higher iron-deficiency anemia and anemia of inflammation compared to settled women. Semi-settled women also had lower intakes of iron. Conversely, Nathan et al. (50) found that Kenyan children aged <6 y living in a nomadic settlement had higher Hb concentrations than children living in agro-pastoral and peri-urban communities. Differences in dietary patterns were also observed; nomadic pastoral children consumed more camel milk and less meat than children living in more settled communities.

The second type of AHC livelihood studies compared AHC-dependent communities with communities reliant predominantly on farming. In the same region of Kenya as the 2 studies above, Miller (49) found that lactating Ariaal pastoral women had higher Hb concentrations compared to women living in communities reliant on a combination of subsistence agriculture, pastoralism, and markets. Livestock units were higher in the pastoral community than the mixed agriculture communities; however, this was not an important predictor of Hb concentrations in adjusted analyses. Jankowska et al. (45) similarly found lower anemia among young Malian children living in livestock livelihoods zones using spatial modeling. In contrast, Keverenge-Ettyang et al. (47) found that Kenyan pastoral women were more anemic in their third trimester of pregnancy and during lactation than farming women. The only study that compared a fishing community with a farming community, conducted by Agyepong et al. (37), reported a lower prevalence of anemia and malaria among Ghanaian school-aged girls living in the fishing community.

### Observational studies of associations between AHC and anemia

Among observational studies that assessed the association between AHC and anemia, we identified mixed results depending on the study population and how AHC predictors were analyzed. Ahenkorah et al. (38) observed that the odds of anemia were twice as high among Ghanaian pregnant women exposed to domestic livestock compared to those not exposed. In contrast, Jones et al. (46) found no association between livestock ownership and anemia among non-pregnant Ghanaian women in models adjusting for sociodemographic characteristics of women, access to improved water and sanitation, and malaria prevention strategies. Jones et al. (46) also analyzed the association of ownership of specific livestock species with anemia among women and found no associations. Conversely, Flores-Martinez et al. (42) found better anemia outcomes among women who lived in sheep- and goat-owning households in Afghanistan, but worse outcomes with chicken ownership, adjusting for household and maternal characteristics, pregnancy status, water source, and region. These authors further examined ASF consumption patterns among sheep-owning households and found that these households were more likely to consume mutton and consume it in greater amounts.

Studies that included children, of which 2 assessed young children while the others included children aged <5 y plus older children, also reported mixed results. Chickens, and no other livestock, were identified as a uniquely important species. Jones et al. (46) found 50% higher odds of anemia among young children from livestock-owning households compared to children from households with no livestock, adjusting for sociodemographic characteristics and malaria parasitemia, among other factors. In analyses examining ownership of individual livestock species, only chicken ownership remained positively associated with anemia. Household ownership of livestock, aggregated and by species, was not associated with ASF consumption in children, nor did ASF consumption mediate the association between livestock ownership and anemia. Iannotti et al. (44) also found a positive association between poultry ownership and severe anemia, adjusting for study design, sex, stunting, and ASF consumption, among boys and girls aged 3–13 y in Haiti. ASF consumption was marginally associated with lower odds of severe anemia in children, but no analysis was conducted of the association between livestock ownership and ASF consumption patterns among the children. In contrast to these 2 studies, in unadjusted analyses, Custodio et al. (40) found a negative association of chicken ownership and severe anemia in young children. Custodio et al. (40) was the only study to include hunting as a predictor of anemia and found a positive association of hunting by a household member and moderate to severe anemia in young children in adjusted analyses.

### AHC interventions

In the AHC intervention studies, impacts on anemia ranged from no effect to modestly reduced anemia in women and children. All but 1 of the studies were integrated interventions that combined education, gardening, supplementation, or multiple of these intervention arms, with animal husbandry. Most interventions focused on increasing poultry production, either alone (52–54) or combined with the production of other small livestock (43, 51, 57, 58) and fish (56, 58), while 1 intervention focused solely on fish production (48).

Six of the 9 intervention studies were evaluations of Helen Keller International Homestead Food Production (HFP) programs in Asia (43, 51–54, 57). The 3 main components of these programs were poultry production, home gardening, and health and nutrition education targeted at women. In repeated cross-sectional analyses of HFP programs implemented in Bangladesh, Cambodia, Nepal, and the Philippines, anemia was reduced among women and young children from beneficiary communities. However, these changes were not statistically different in magnitude compared to those in non-participating communities (43, 57). A 2-y randomized controlled trial conducted in Burkina Faso found a decline in the prevalence of anemia, from 90.3% to 76.8%, among young children aged 3–5.9 mo whose mothers had received an HFP plus health behavior change intervention. This decline was in comparison to the increased prevalence of anemia among young children in control villages (from 85.7% to 86.2%) (52). An 11-mo evaluation of a trial conducted in Nepal found greater reductions in anemia among young children receiving HFP and HFP plus micronutrient powder supplementation interventions compared to young children in control villages (48.6 and 51.5 percentage point reduction compared with 39.6 percentage point reduction) (53). Subsequent analyses of this randomized controlled trial found that, after 2.5 y, children 12–48 mo participating in the HFP intervention had 24% lower adjusted odds of anemia and women 38% lower adjusted odds of anemia compared with children and women, respectively, in the control group (54).

One of the pathways by which the Helen Keller International HFP programs aim to reduce anemia is by increasing consumption of ASF through increased poultry production. In some of the HFP programs, egg consumption increased among children and women (43, 51, 57), and chicken liver consumption increased among households (57). Of the HFP evaluations that assessed morbidity symptoms, 1 study in Cambodia (51), but not another in Nepal (53), found a lower prevalence of fever in children, while 2 studies in Burkina Faso and Nepal observed reductions in diarrhea among children (52, 53).

Three studies assessed the impact of fish pond production interventions on anemia (48, 56, 58). A school-based intervention in Thailand that promoted fish ponds, poultry production, vegetable gardening, nutrition education, improved school lunches, and iron supplementation, found no impact on Hb among girls aged 10-13 y in the intervention group compared with those in the comparison group, despite an increase in iron intake from dietary sources and supplementation (56). In contrast, a multiple-component nutrition improvement program in China that included promotion of freshwater aquaculture found a 49.3 percentage point reduction in anemia prevalence among young children (58). Lastly, a fishpond intervention conducted in Bangladesh reduced the prevalence of anemia after 10 y among women whose households received private and group-operated polyculture fishpond technology (29.5 and 8.2 percentage point reductions, respectively), compared to almost no change among women in the vegetable production group (0.2 percentage point increase) (48).

### Risk of bias within studies and summary of evidence

There is a serious risk of bias within most studies that were evaluated in this review, with fewer than one-quarter being judged as having “low risk of bias” (Supplemental Table 2). Studies with a low risk of bias compared households engaged in AHC to households without animals, while also controlling for confounding variables. These studies also included analyses of livestock by species or by quantity of livestock. Although several studies measured ASF consumption or iron intake, few assessed morbidity outcomes, and of those that did, the majority measured self-reported illness. Only 1 study used appropriate biomarkers to differentiate anemia caused by iron deficiency versus inflammation, whereas the rest relied predominantly on Hb. Furthermore, most studies were observational, and the few intervention studies that had a robust study design did not include a treatment arm to evaluate the independent effect of AHC.

Taking into account the risk of bias within each study, the overall quality of evidence to draw conclusions about the association between AHC and anemia outcomes in each population group is poor (**Table 2**). The most commonly analyzed AHC exposures were pastoral livelihoods among women and animal production interventions among women and young children. Fewer than 2 studies per population studied fishing and hunting exposures, and few studies included school-aged children. After excluding studies with a “high risk of bias,” animal production interventions among young children had the strongest literature base to assess an effect of AHC on anemia outcomes. Overall, these interventions had a beneficial effect on anemia. However, based on the risk of bias assessment, these studies were mostly “unclear” in risk of bias because of a multiple-treatment arm design without an individual effect assessment of animal production on anemia. Among lower risk observational studies, a consistent result was identified among young children for both the pastoral livelihood exposure (beneficial) and household chicken ownership (harmful), although this evidence comes from only 2 studies within each exposure.

**TABLE 2:** Summary of evidence indicating positive (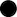), null (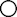), or negative (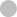) anemia outcomes among women of reproductive age, school-aged children, and children aged <5 y by AHC exposures within reviewed studies^1^

AHC exposure	Anemia outcomes (within all studies)^2^	Anemia outcomes (excluding high bias studies)^3^	Summary of evidence (excluding high bias studies)^3^^,^^4^
Women of reproductive age			
Pastoral livelihood	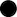 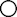 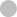 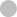 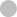	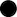 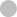	Positive/negative
Livestock ownership (aggregated)	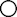 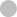	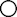 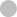	NA/negative
Chickens	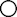 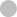	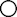 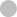	NA/negative
Goats	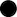 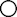	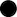 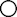	NA/positive
Sheep	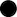 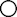	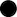 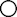	NA/positive
Cattle	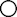 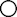	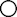 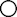	NA
Animal production intervention	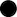 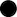 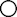 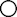	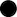 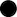 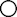	Positive
Fish intervention	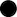	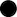	Positive
School-aged children			
Fishing livelihood	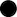	−	Unclear
Livestock ownership (aggregated)	−	−	−
Chickens	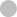	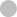	Negative
Fish/animal production intervention	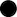	−	Unclear
Children aged <5 y			
Pastoral livelihood	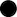 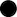 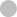	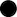 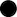	Positive
Livestock ownership (aggregated)	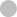	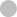	Negative
Chickens	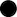 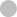 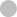	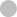 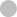	Negative
Goats	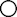	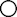	NA
Sheep	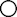	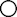	NA
Cattle	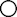	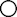	NA
Hunting by household member	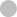	−	Unclear
Fish/animal production	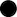 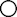	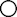	NA
Animal production intervention	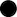 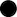 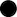 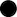 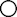 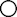	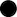 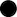 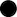 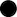 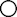	Positive

1 AHC, animal husbandry and capture; Hb, hemoglobin; NA, no association.

2 A single study may be listed more than once if the authors analyzed multiple AHC exposures separately.

3 Includes only those studies judged as having “low” or “unclear” risk of bias based on the risk of bias assessment (Supplemental Table 2).

4 Positive indicates better anemia status (lower anemia or higher Hb concentration in the exposed versus comparison population), negative indicates worse anemia status, NA indicates no association, and unclear indicates insufficient evidence to determine an overall effect. Significance values from individual studies were not considered for the summary of evidence assessment, but rather this evidence assessment reflects a general trend of association between the respective AHC exposures and anemia.

## Conclusions

This systematic review assessed the relation between animal husbandry and capture and anemia among women of reproductive age, school-aged children, and children aged <5 y by evaluating evidence from observational and intervention studies of pastoral livelihoods, livestock ownership, and fishing. A secondary objective of the review was to explore the hypothesized pathways by which AHC may influence anemia status as shown in Figure 1 (i.e., through ASF consumption and pathogenic infections). Overall, we found little consistency in results across several AHC exposures and population groups. Therefore, we have limited confidence in determining whether AHC promotes or prevents anemia. Further, our assessment of the evidence was hampered by study methods that were not designed to answer our research questions; for example, studies had poor comparison groups, missing data on diet and morbidity indicators, and insufficient adjustment for confounding. Our conclusions agree with 2 other recent reviews that the evidence base for evaluating iron and anemia status, consumption of ASF, and morbidity outcomes as they relate to animal production is weak (22, 23). Nevertheless, we noted that some AHC exposures were more often associated with specific anemia outcomes. Below, we elaborate on these trends and describe recommendations and implications for future research.

In general, women from nomadic pastoral communities were more often anemic than settled pastoral or farming women, whereas, in contrast, anemia was less common among young children living nomadic pastoral livelihoods. Milk, and to a lesser extent meat, is an important dietary component for nomadic populations (59) and has been linked to improved nutritional status in studies comparing nomadic children to children in settled communities (60). Studies of shifts towards sedentarization among pastoral communities have consistently found greater malnourishment among sedentarized children and women, linked to increased consumption of cereals and sugar and decreased consumption of milk (61, 62). Although milk is a vital source of energy for nomadic pastoralists, it is not an important dietary source of iron (11), and the high calcium content in milk may inhibit iron absorption (63). Thus, it is unlikely that shifts in milk consumption underlie differences in anemia among nomadic and settled pastoral groups. Further, among the reviewed studies, groups with higher prevalences of morbidity tended to have worse anemia status, which may indicate the importance of infections and illness in determining anemia status among these populations, regardless of the degree of sedentarization. Given this, it is more likely that other health factors resulting from shifts in livelihoods impact anemia status among pastoralist populations, which limits the strength of this evidence to understand how living in proximity to animals affects anemia.

Both women and young children benefited from interventions that combined animal production with education and home gardening components. However, it was not possible to disentangle the effects of animal production from benefits received by improved knowledge of young child feeding practices, improved maternal health practices, and access to a greater diversity of foods from home gardening. Moreover, improvements in the intervention groups tended to be both modest and non-significant, with some interventions finding worse anemia status at endline than baseline, albeit better than control groups. Most interventions were successful in increasing household poultry and egg production, as well as egg consumption among women and children. The studies that measured morbidity indicators found lower prevalences of diarrhea and fever among young children in intervention groups, but this likely reflects improved sanitation and hygiene practices (54), rather than changes in animal production. However, the potential importance of sanitation and hygiene in reducing the burden of infectious disease from animals remains inadequately explored (27, 64), and recent water, sanitation, and hygiene interventions have had limited impacts on diarrhea and nutritional outcomes (65, 66). Further, assessment of diarrhea provides no quantification of pathogen burden, may be subject to recall bias (67), and does not provide information on asymptomatic enteric infections (68). Unfortunately, none of these intervention studies directly measured pathogenic infections that could contribute to anemia.

It is notable that poultry production is the predominant animal component promoted in intervention studies, yet, in observational studies, chicken ownership was positively associated with anemia in young children. Unlike larger livestock species, chickens are readily accessible, affordable, and can be managed and sold by women (69), and thereby are an ideal animal to rear in low-income settings. However, recent work has found that keeping poultry indoors is associated with growth faltering in young children (70), likely mediated through exposure to pathogens in feces. Chicken feces contain large amounts of pathogenic bacteria such as *Campylobacter jejuni* and *Escherichia coli* that can contaminate the home environment where children eat and play (25, 71), can be ingested if children unknowingly touch feces (72, 73), and can contribute to enteropathogen infection in children (18, 27, 74). High enteropathogen burden is a significant risk factor for stunting in children aged <2 y (75), and repeated exposure to pathogens from feces may contribute to a subclinical inflammatory condition known as environmental enteric dysfunction, which is characterized by villous blunting, intestinal inflammation, and malabsorption (76). Through similar mechanisms, repeated bacterial infections from exposure to chicken feces might also contribute to the pathogenesis of anemia by promoting intestinal inflammation and malabsorption that impairs iron absorption (77) as well as by inducing hepcidin secretion (78), which would further inhibit dietary iron absorption and induce iron sequestration into macrophages and hepatocytes (79). This hypothesized mechanism may explain why some studies in our review found worse anemia outcomes among children living in households that own poultry.

Many studies in this review assessed ASF consumption to determine how differences in dietary intake correlated with anemia status among comparison groups. ASF consumption tended to be higher among households that engaged in animal rearing, particularly for chicken meat and egg consumption among poultry-owning households. However, we found insufficient evidence in our review to determine whether ASF consumption from AHC is associated with lower anemia. Furthermore, livestock production may not always increase ASF consumption if animals are used for income rather than food (80), or if the intra-household allocation of ASF is unequal among household members (81, 82). Alternatively, anemia in these settings may not only be caused by insufficient iron in the diet, but also acute infections or chronic inflammation from exposure to pathogens. Unfortunately, of the few studies that assessed the prevalence of malaria or helminth infection, none analyzed the association of these pathogenic infections directly with AHC. Further, no studies measured enteropathogenic bacteria. Thus, the mechanisms by which AHC may impact anemia remain poorly understood.

Future research is needed to adequately analyze the complex and potentially contradictory pathways that connect AHC and anemia. Our understanding of these pathways would be improved by detailed assessment of ASF intake, including distinguishing ASF derived from own-production versus purchases, and quantification of pathogens in feces and other fomites that can be transmitted from livestock to humans. More robust measures of livestock ownership and anemia are also needed. For example, because of the potential importance of certain livestock species (i.e., chickens) and livestock management practices (e.g., corralling versus free-roaming) in determining disease risk among young children, assessment of livestock ownership should include data on livestock species and quantity as well as management. Additionally, given the complex etiology of anemia, assessment of only Hb is insufficient to understand the predominant drivers of anemia in a given context (e.g., nutritional deficiencies in comparison to infection). Future studies would benefit from including iron status and inflammatory biomarkers (i.e., serum ferritin, serum transferrin receptor, C-reactive protein, α-1-acid glycoprotein) that can distinguish anemia caused by iron-deficiency or inflammation, as recommended by the BRINDA (Biomarkers Reflecting Inflammation and Nutritional Determinants of Anemia) project (83).

Animals hold an essential place in our food system and understanding their role in different contexts is integral to meeting several of the UN Sustainable Development Goals, particularly ending hunger and poverty while also preserving natural resources (84). The nutrition and income derived from AHC may be important in reducing malnutrition among vulnerable populations, especially young children, adolescent girls, and women, but careful assessment of potential harm from animal production is needed as we continue to promote small-scale animal husbandry, especially of chickens, among poor households. In addition to understanding the immediate consequences of AHC in such contexts, future research must also emphasize understanding of potential long-term adverse effects of increased animal production and consumption on non-communicable disease risks, such as cancer and cardiovascular disease risk, and impacts on climate, natural resources, and biodiversity. Health, environmental, and ethical considerations of AHC warrant the need to investigate appropriate animal production strategies that promote human, animal, and environmental health.

## Supplementary Material

Supplement File

## Notes

Supported in part by the Bill and Melinda Gates Foundation (OPP1154099).

Author disclosures: NJL, MLW, and ADJ, no conflicts of interest.

Supplemental Tables 1–3 are available from the “Supplementary data” link in the online posting of the article and from the same link in the online table of contents at https://academic.oup.com/advances/.

Abbreviations used: AHC, animal husbandry and capture; ASF, animal source foods; GRADE, Grading of Recommendations, Assessment, Development, and Evaluation; Hb, hemoglobin; HFP, Homestead Food Production; LMICs, low- and middle-income countries.
