# Supplementary material for: Assessing the Impact of Animal Husbandry and Capture on Anemia among Women and Children in Low- and Middle-Income Countries: A Systematic Review
Source: Adv Nutr. 2019 Mar 11;10(2):331–44. doi: 10.1093/advances/nmy080 (PMC6416043; doi:10.1093/advances/nmy080)
Supplement: Supplement File [file nmy080_supplemental_file.pdf]

Supplemental Table 1: Electronic search strategy.

Database (URL)	Search Terms	Number of Hits	Notes
Pubmed (https://www.ncbi.nlm.nih.gov/pubmed)	(“animal husbandry”[MeSH Terms] OR “livestock production” OR livestock OR cow OR chicken OR poultry OR goat OR “sheep” OR mutton OR camel OR pig OR "small ruminant" OR "dairy production" OR "milk production" OR "egg production" OR pastoral OR transhumant OR transhumance OR aquaculture OR aqua-culture OR “small-scale fishery” OR “small-scale fisheries” OR “fish farm” OR “fish production” OR “small fish species” OR fishing OR wild meat OR bush meat OR “bushmeat”) AND (anemia OR anaemia OR “iron deficiency” OR “iron-deficiency” OR “iron status” OR “iron-status” OR “hemoglobin” OR “haemoglobin” OR “animal source food consumption” OR “animal-source food consumption” OR “dietary diversity” OR “micronutrient status” OR vitamin B12 OR vitamin B-12 OR retinol OR vitamin A OR “folate” OR “folic acid” OR iron OR zinc OR “environmental enteric dysfunction” OR “tropical enteropathy” OR “environmental enteropathy” OR “zoonotic infection” OR “zoonoses” OR parasitic infection OR parasitic disease OR infectious disease OR “vector-borne illness” OR “vector-borne disease” OR helminth infection OR (“women’s” AND (“labor” OR “labour” OR “work” OR “income” OR “expenditure” OR “livelihood” OR “role” OR “empowerment” OR “time” OR “decision making”))) AND (woman[Title/Abstract] OR women[Title/Abstract] OR adolescent[Title/Abstract] OR child[Title/Abstract] OR children[Title/Abstract] OR maternal health[Title/Abstract] OR household[Title/Abstract])	2,246	Search “All Fields.”
Web of Science (https://webofknowledge.com/)	(“animal husbandry” OR “livestock production” OR livestock OR cattle OR cow OR chicken OR poultry OR goat OR sheep OR mutton OR camel OR pig OR swine OR "small ruminant" OR "dairy production" OR “dairy promotion” OR "milk production" OR “milk promotion” OR "egg production" OR “egg promotion” OR pastoral OR pastoralism OR pastoralist OR transhumant OR transhumance OR aquaculture OR aqua-culture OR “small-scale fishery” OR “small-scale fisheries” OR “fish farm” OR “fish production” OR “small fish species” OR “fishing” OR “wild meat” OR “bush meat” OR “bushmeat”) AND (anemia OR anaemia OR “iron deficiency” OR “iron-deficiency” OR “iron status” OR “iron-status” OR “hemoglobin” OR “haemoglobin” OR “animal source food consumption” OR “animal-source food consumption” OR “dietary diversity” OR “micronutrient status” OR “vitamin B12” OR “vitamin B-12” OR retinol OR vitamin A OR folate OR “folic acid” OR iron OR zinc OR “environmental enteric dysfunction” OR “tropical enteropathy” OR “environmental enteropathy” OR “zoonotic infection” OR zoonoses OR “zoonotic disease” OR “parasitic infection” OR “parasitic disease” OR “infectious disease” OR “vector-borne illness” OR “vector-borne disease” OR “helminth infection” OR helminthiasis OR “women’s labor” OR “women’s labour” OR “women’s work” OR “women’s income” OR “women’s expenditure” OR “women’s livelihood” OR “women’s role” OR “women’s empowerment” OR “women’s time” OR “women’s decision making”) AND (woman OR women OR adolescent OR child OR children OR “maternal health” OR household)	1,855	Search “Topic.”

EMBASE (www.embase.com)	(‘animal husbandry’/exp OR ‘animal husbandry’ OR ‘livestock production’ OR ‘livestock’/exp OR livestock OR cow OR cattle OR chicken OR poultry OR goat OR sheep OR mutton OR camel OR pig OR swine OR ‘small ruminant’ OR ‘dairy production’ OR ‘milk production’ OR ‘egg production’ OR pastoral OR pastoralism OR pastoralist OR transhumant OR transhumance OR aquaculture OR aqua-culture OR ‘small-scale fishery’ OR ‘small-scale fisheries’ OR ‘fish farm’ OR ‘fish production’ OR ‘small fish species’ OR fishing OR ‘wild meat’ OR ‘bush meat’ OR ‘bushmeat’) AND (anemia/exp OR anemia OR anaemia OR ‘iron deficiency’/de OR ‘iron deficiency’ OR ‘iron-deficiency’ OR ‘iron status’ OR ‘iron-status’ OR ‘hemoglobin’ OR ‘haemoglobin’ OR ‘animal source food consumption’ OR ‘animal-source food consumption’ OR ‘dietary diversity’ OR ‘micronutrient status’ OR ‘vitamin B12’ OR ‘vitamin B-12’ OR retinol OR ‘vitamin A’ OR folate OR ‘folic acid’ OR iron OR zinc OR ‘environmental enteric dysfunction’ OR ‘tropical enteropathy’ OR ‘environmental enteropathy’ OR ‘zoonotic infection’ OR ‘zoonoses’ OR parasitosis/exp OR ‘parasitic infection’ OR ‘parasitic disease’ OR ‘infectious disease’ OR ‘vector-borne illness’ OR ‘vector-borne disease’ OR ‘helminth infection’ OR helminthiasis OR 'women/s labor' OR 'women/s labour' OR 'women/s work' OR 'women/s income' OR 'women/s expenditure' OR 'women/s livelihood' OR 'women/s role' OR 'women/s empowerment' OR 'women/s time' OR 'women/s decision making') AND (woman:ti,ab OR women:ti,ab OR adolescent:ti,ab OR child:ti,ab OR children:ti,ab OR maternal health:ti,ab OR household:ti,ab)	997	Search “All fields.” No mapping options selected.
Global Health (https://www.ebscohost.com/academic/global-health)	(“animal husbandry” OR “livestock production” OR livestock OR cattle OR cow OR chicken OR poultry OR goat OR sheep OR mutton OR camel OR pig OR swine OR "small ruminant" OR "dairy production" OR “dairy promotion” OR "milk production" OR “milk promotion” OR "egg production" OR “egg promotion” OR pastoral OR pastoralism OR pastoralist OR transhumant OR transhumance OR aquaculture OR aqua-culture OR “small-scale fishery” OR “small-scale fisheries” OR “fish farm” OR “fish production” OR “small fish species” OR fishing OR wild meat OR bush meat OR bushmeat) [TX All Text] AND (anemia OR anaemia OR “iron deficiency” OR “iron-deficiency” OR “iron status” OR “iron-status” OR “hemoglobin” OR “haemoglobin” OR “animal source food consumption” OR “animal-source food consumption” OR “dietary diversity” OR “micronutrient status” OR “vitamin B12” OR “vitamin B-12” OR retinol OR vitamin A OR folate OR “folic acid” OR iron OR zinc OR “environmental enteric dysfunction” OR “tropical enteropathy” OR “environmental enteropathy” OR “zoonotic infection” OR zoonoses OR “zoonotic disease” OR “parasitic infection” OR “parasitic disease” OR “infectious disease” OR “vector-borne illness” OR “vector-borne disease” OR “helminth infection” OR helminthiasis OR “women’s labor” OR “women’s labour” OR “women’s work” OR “women’s income” OR “women’s expenditure” OR “women’s livelihood” OR “women’s role” OR “women’s empowerment” OR “women’s time” OR “women’s decision making”) [TX All Text] AND (woman OR women OR adolescent OR child OR children OR “maternal health” OR household) [AB Abstract]	3,557	Advanced Search: Chunk 1 as “TX All Text” AND Chunk 2 as “TX All Text” AND Chunk 3 as “AB Abstract” (as shown in Search Terms).

Supplemental Table 2: Risk of bias in individual studies[1].

Study Author (Ref)	Counterfactual Assessment	Sample Size	Anemia Outcome Assessment	Intermediate Outcome Assessment	Confounding Bias Assessment	Overall Judgment
Adongo et al. (36)	Yes (measured TLU)	Unclear (N of blood sampling justified as representative of sample population, but no N calculation)	Yes (HemoCue)	Yes (24h dietary recall, inflammatory biomarkers - CRP, AGP, TfR)	No (no adjusted analyses)	Unclear risk of bias
Agyepong et al. (37)	No (limited description of AHC to draw comparisons)	Unclear (lack of information)	Yes (cyanide methaemoglobin method)	Yes (malaria, self-reported morbidity symptoms)	No (no adjusted analyses)	High risk of bias
Ahenkorah et al. (38)	Yes (presence of domestic livestock)	Unclear (lack of information)	Yes (Sysmex KX-21N Automated Haematology Analyzer)	No (malaria, enteric parasites, *Schistosomiasis* collected but no association measured with AHC)	Yes (regression with maternal age, urban, education, occupation, gravidity, parity, trimester of pregnancy, water source, previous history of anemia, and presence of domestic livestock variables)	Low risk of bias
Bechir et al. (39)	Unclear (limited description of AHC, no quantification)	No (sample size powered for Vitamin A as outcome)	Yes (HemoCue)	Yes (intestinal parasites, malaria)	No (adjusted analyses conducted for malnutrition as outcome)	High risk of bias
Custodio et al. (40)	Yes (measured livestock ownership by species, hunting, and fishing)	Unclear (lack of information)	Yes (hematocrit)	No (malaria and morbidity symptoms collected but no associations measured with AHC)	Unclear (adjusted analyses conducted but data not shown)	High risk of bias

Study Author (Ref)	Counterfactual Assessment	Sample Size	Anemia Outcome Assessment	Intermediate Outcome Assessment	Confounding Bias Assessment	Overall Judgment
Dalsin (41)	Unclear (all regions dependent on AHC, but distinctions by livestock type and quantity)	Unclear (lack of information)	Yes (HemoCue)	Yes (diet)	No (no adjusted analyses)	High risk of bias
Flores-Martinez et al. (42)	Yes (livestock ownership by species)	N/A (analysis of secondary data set)	Yes (HemoCue)	Yes (diet, food sourcing)	Yes (altitude adjusted and unadjusted regression with age, education, HH head education, pregnancy, birth in last two years, 3+ children, number of HH members, number of under-5s in household, language/ethnicity, wealth quintile, treated drinking water, electricity, agricultural land, HH owns cattle, HH owns horses/donkeys, HH owns goats, HH owns sheep, HH owns chicken, in rural area, region variables)	Low risk of bias
Hillenbrand and Waid (43)	Unclear (combined intervention with no separation of poultry component)	Unclear (lack of information)	Yes (HemoCue)	Unclear (diet, from supplemental report)	Unclear (difference in difference estimates)	Unclear risk of bias

Study Author (Ref)	Counterfactual Assessment	Sample Size	Anemia Outcome Assessment	Intermediate Outcome Assessment	Confounding Bias Assessment	Overall Judgment
Iannotti et al. (44)	Yes (livestock ownership by species)	Unclear (lack of information)	Yes (HemoCue)	No (diet and morbidity collected but no associations measured with AHC)	Yes (regression with age of child, sex of child, stunted, vitamin A supplement in last 6mo, ASF consumption in last 24h, deworming in last 6mo, fever in last two weeks, poultry ownership variables)	Unclear risk of bias
Jankowska et al. (45)	Unclear (livelihood zones described by livestock rearing but no quantification, HHs clustered into zones and not independently assessed)	N/A (analysis of secondary data set)	Yes (HemoCue)	No (assess climate as a determinant)	Yes (regression with livelihood zone, age of child, children ever born per mother, age of HH head, HH wealth, unprotected well variables)	Unclear risk of bias
Jones et al. (46)	Yes (livestock ownership by species)	N/A (analysis of secondary data set)	Yes (HemoCue)	Yes (diet and HH food consumption/expenditu re on ASF)	Yes (regression with HH livestock ownership, HH size, age (years or months), sex of HH head, sex of child, education of woman/mother, urban, wealth quintiles, improved water source, sanitation source, anti-malaria indoor spraying, mosquito bed net, current tobacco use, weight status, parity, fever in previous two weeks, diarrhea in previous	Low risk of bias

Study Author (Ref)	Counterfactual Assessment	Sample Size	Anemia Outcome Assessment	Intermediate Outcome Assessment	Confounding Bias Assessment	Overall Judgment
					two weeks, consumption of vitamin A supplement in previous 6mo, consumption of iron supplement in previous 7 days, treatment for intestinal worms in last 6mo, breastfeeding status, presence of malaria parasites variables)	
Keverenge-Ettyang et al. (47)	Unclear (no quantification of AHC)	Unclear (lack of information)	Yes (Coulter counter method)	No (no measures of diet or morbidity, measured ferritin for iron stores)	No (no adjusted analyses)	High risk of bias
Kumar and Quisumbing (48)	Yes (fishpond interventions and vegetable gardening intervention comparison, non-randomized)	Unclear (lack of information)	Yes (methodology information lacking)	Yes (diet)	Unclear (difference in difference estimates)	Low risk of bias
Miller (49)	Yes (description of AHC and quantification of TLU)	Unclear (lack of information)	Yes (HemoCue)	Yes (self-reported illness)	Yes (regression with age, parity, months since birth, returned to menstruation, upper arm fat area, livestock units, community, reported illness variables)	Low risk of bias

Study Author (Ref)	Counterfactual Assessment	Sample Size	Anemia Outcome Assessment	Intermediate Outcome Assessment	Confounding Bias Assessment	Overall Judgment
Nathan et al. (50)	Yes (description of AHC, TLU measured but not reported)	Unclear (lack of information)	Yes (HemoCue)	Yes (24h dietary recall, maternal-reported morbidity symptoms)	No (no adjusted analyses)	Unclear risk of bias
Olney et al. (51)	Unclear (combined intervention with no separation of poultry component, assessment of animal production by species, non-randomized)	Unclear (lack of information)	Yes (HemoCue)	Yes (dietary intake survey, reported morbidity symptoms)	No (no adjusted analyses with Hb/anemia as outcome)	Unclear risk of bias
Olney et al. (52)	Unclear (combined intervention with no separation of poultry component, no quantification of AHC, randomized)	Yes	Yes (HemoCue)	Yes (dietary diversity, maternal-reported diarrhea)	Unclear (difference in difference estimates)	Unclear risk of bias
Osei et al. (53)	Unclear (combined intervention with no separation of poultry component, quantification of poultry at baseline, randomized)	Yes	Yes (HemoCue)	Yes (maternal-reported morbidity symptoms	Yes (regression with intervention group, age, sex, baseline anemia, HAZ, WHZ, mother's education, HH wealth)	Unclear risk of bias
Osei et al. (54)	Unclear (combined intervention with no separation of poultry component, data not shown on quantification of poultry, randomized)	Unclear (sample size powered for difference in prevalence of stunting, underweight, and wasting among children)	Yes (HemoCue)	Unclear (complementary feeding practices but no ASF-specific measure, infection data collected but not shown)	Yes (regression with treatment, time, cluster design, age of child, sex of child, parity, pregnancy status, education of mother, HH size, gender of HH head, marital status of	Unclear risk of bias

Study Author (Ref)	Counterfactual Assessment	Sample Size	Anemia Outcome Assessment	Intermediate Outcome Assessment	Confounding Bias Assessment	Overall Judgment
					HH head caste of family)	
Schipani et al. (55)	Unclear (fishpond and small-animal husbandry combined with gardening with non-gardening comparison, quantification of AHC at baseline)	Unclear (lack of information)	Yes (HemoCue)	Yes (24h dietary recall, ferritin)	No (no adjusted analyses)	Unclear risk of bias
Smitasiri and Dhanamitta (56)	No (combined intervention with no separation of AHC components, no quantification of AHC, non-randomized)	Unclear (sample size powered for change in serum retinol)	Yes (cyanmethemoglobin method)	Yes (24h dietary recall, ferritin)	No (no adjusted analyses)	High risk of bias
Talukder et al. (57)	Unclear (combined intervention with no separation of poultry component, no quantification of AHC, non-randomized)	Unclear (lack of information)	Yes (HemoCue)	Yes (diet - ASF consumption)	No (no adjusted analyses)	High risk of bias
Wang et al. (58)	No (no comparison group)	Unclear (lack of information)	Yes (cyanmethemoglobin method)	No (ASF/iron intake data at baseline, no data at endline)	No (no adjusted analyses)	High risk of bias

1. Abbreviations: AGP – α-1-acid glycoprotein, AHC – animal husbandry and capture, ASF – animal source foods, CRP – C-reactive protein, HAZ – height-for-age Z-score, HH – household, TfR – transferrin receptor, TLU – tropical livestock units, WHZ – weight-for-height Z-score

Supplemental Table 3: Criteria for assessing risk of bias in individual studies[1].

Criteria	Description	Weighting[2]
**Counterfactual Assessment**	Were comparison groups appropriately selected to allow counterfactual analysis for the measurement of AHC? Supporting evidence: description of AHC livelihoods, quantification of number of livestock, description/quantification of different species of livestock.	***
**Sample Size**	Was an appropriate sample size justified to detect differences in the outcome (i.e., anemia)?	*
**Outcome Assessment**	Was the outcome measure (i.e., hemoglobin, anemia) appropriately assessed by the investigators (quantified by lab assay/Hemocue vs. self-report) and adjusted as necessary (e.g., altitude)?	*
**Intermediate Outcome Assessment**	Were one or more intermediate outcomes (i.e., diet, morbidity) assessed to explain observed differences by AHC?	**
**Confounding Assessment**	Were appropriate statistical analyses conducted to control for key confounding variables (e.g., malaria, age, pregnancy status, wealth index) in assessing the relationship between AHC and anemia?	***

1. Abbreviations: AHC – animal husbandry and capture
2. Weighting scale: *** most important to *least important
